# Magneto-Transport in Flexible 3D Networks Made of Interconnected Magnetic Nanowires and Nanotubes

**DOI:** 10.3390/nano11010221

**Published:** 2021-01-16

**Authors:** Tristan da Câmara Santa Clara Gomes, Nicolas Marchal, Flavio Abreu Araujo, Yenni Velázquez Galván, Joaquín de la Torre Medina, Luc Piraux

**Affiliations:** 1 Institute of Condensed Matter and Nanosciences, Université Catholique de Louvain, Place Croix du Sud 1, 1348 Louvain-la-Neuve, Belgium; tristan.dacamara@uclouvain.be (T.d.C.S.C.G.); nicolas.marchal@uclouvain.be (N.M.); flavio.abreuaraujo@uclouvain.be (F.A.A.); yenni.velazquez.g@gmail.com (Y.V.G.); 2 Instituto de Investigaciones en Materiales-Unidad Morelia, Universidad Nacional Autónoma de México, Morelia 58000, Mexico; delatorre@materiales.unam.mx

**Keywords:** magnetic nanostructures, 3D nanowire networks, 3D nanotube networks, anisotropic magnetoresistance, 3D nanomagnetism

## Abstract

Electrochemical deposition of interconnected nanowires and nanotubes made of ferromagnetic metals into track-etched polycarbonate templates with crossed nanochannels has been revealed suitable for the fabrication of mechanically stable three-dimensional magnetic nanostructures with large surface area. These 3D networks embedded into flexible polymer membranes are also planar and lightweight. This fabrication technique allows for the control of the geometric characteristics and material composition of interconnected magnetic nanowire or nanotube networks, which can be used to fine-tune their magnetic and magneto-transport properties. The magnetostatic contribution to the magnetic anisotropy of crossed nanowire networks can be easily controlled using the diameter, packing density, or angle distribution characteristics. Furthermore, the fabrication of Co and Co-rich NiCo alloy crossed nanowires with textured hcp phases leads to an additional significant magnetocrystalline contribution to the magnetic anisotropy that can either compete or add to the magnetostatic contribution. The fabrication of an interconnected nanotube network has also been demonstrated, where the hollow core and the control over the tube wall thickness add another degree of freedom to control the magnetic properties and magnetization reversal mechanisms. Finally, three-dimensional networks made of interconnected multilayered nanowire with a succession of ferromagnetic and non-magnetic layers have been successfully fabricated, leading to giant magnetoresistance responses measured in the current-perpendicular-to-plane configuration. These interconnected nanowire networks have high potential as integrated, reliable, and stable magnetic field sensors; magnetic devices for memory and logic operations; or neuromorphic computing.

## 1. Introduction

Recent developments in the synthesis of three-dimensional (3D) networks made of interconnected nanowires (NWs) and nanotubes (NTs) offer new perspectives for various nanodevices and nano-electronics [1,2,3,4,5,6,7,8] for applications such as energy harvesting and storage systems [3,6,9], sensing devices and actuators [4,7], catalysts [1], electrochromic elements [10], solar cells [11], biosensors and bio-analytical devices [12,13], and spin caloritronics devices [14,15]. Their unique architecture offers advantageous mechanical and geometrical properties such as a good mechanical stability of the self-standing nanostructures, high surface over volume ratio, and lightness to the network films. Moreover, the branching structure leads to an excellent electrical and thermal connectivity, which facilitates the ability to carry out transport measurements of NW and NT nanostructures [16,17,18]. The transport properties can be measured in the plane direction of the network films, along the macroscopic dimensions, leading to reliable measurements of electrical, thermal, and thermoelectric properties [14,16,17,19]. Direct electrochemical deposition into track-etched polymeric membranes has been proved to be a versatile and reliable method for the large-area fabrication of 3D networks [1,8,20]. It allows for controllable size, morphology, NWs or NTs density, and material composition, providing a suitable pathway to fabricate various complex 3D networks of high aspect ratio nanostructures. In addition, magnetic and non-magnetic layers can be electrochemically stacked to obtain interconnected multilayered NW networks [14,21,22] by adapting the pulse electrodeposition technique [23,24,25]. In the context of recent researches for the realization of light-weight, flexible, shapeable, and even wearable magneto-electronic devices [26,27,28,29,30] and thermoelectric devices [30], the polymer membrane also provides flexible and shapeable properties to the network–polymer composite, as well as a protection against the oxidation of metallic NWs or NTs. Here, we report on the synthesis and characterization of the magneto-transport properties of various networks made of NWs, NTs and multilayers fabricated by template-assisted electrodeposition using track-etched polycarbonate (PC) membranes with crossed nanochannels. The magneto-transport properties of NWs and NTs have been intensively studied in single NW [31,32], NWs arranged in parallel arrays [33,34,35], as well as recently in interconnected NW networks [16,17,18,36], in particular to assess the magnetic and magnetization reversal mechanism properties of those nanostructures. Moreover, multilayered NWs have been proved suitable to reliably measured the giant magnetoresistance in the current-perpendicular-to-plane (CPP) configuration, leading to large giant magnetoresistance ratios and reliable measurement of key spintronics parameters [24,37].

## 2. Fabrication

**Figure 1:**
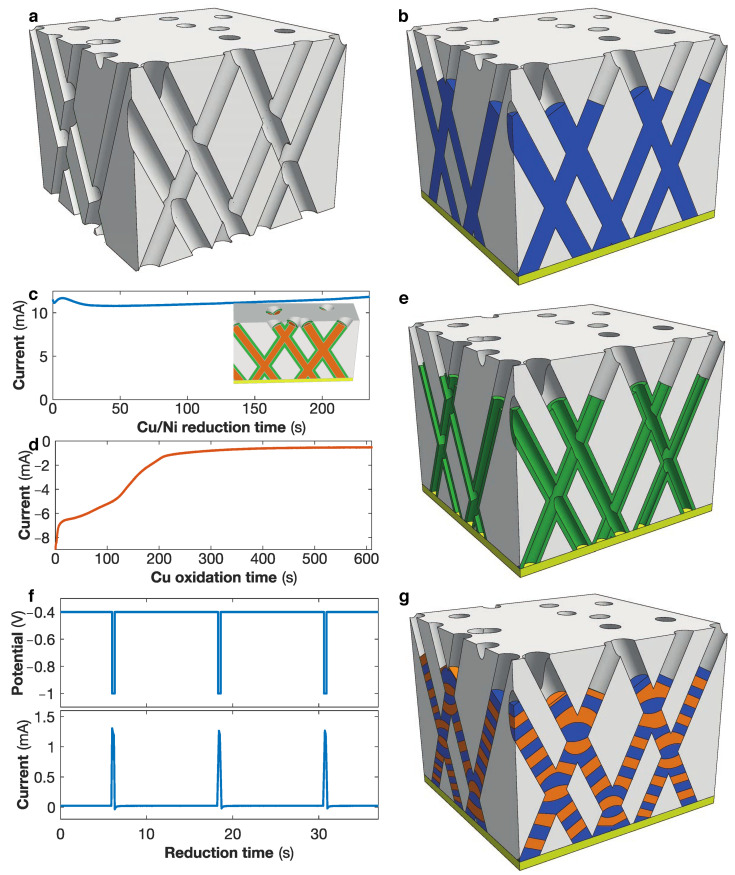
Schematics of (**a**) a polycarbonate template with 3D crossed nanopore network and (**b**) an interconnected nanowire network. (**c**,**d**) Fabrication of interconnected Ni nanotube networks. (**c**) Growth of interconnected Cu/Ni core/shell nanocable networks at constant potential of −1 V and (**d**) dealloying process to selectively etch the Cu core at constant potential +0.2 V. The inset in panel (**c**) shows a schematic of a crossed Cu/Ni core/shell nanocable network. (**e**) Schematic of a crossed Ni nanotube network. (**f**) Fabrication of interconnected multilayered nanowire networks using a pulse electrodeposition technique to successively deposited the ferromagnetic and non-magnetic metal layers. (**g**) Schematic of a crossed nanowire network with a succession of ferromagnetic and non-magnetic layers.

3D NW and NT networks were grown by direct electrodeposition into track-etched polycarbonate membranes with well-defined 3D interconnected cylindrical nanochannel networks. The selected 3D nanoporous templates were fabricated by exposing 22 μm thick PC films using a multi-step track-etching technique at various angles with respect to the out-of-plane (OOP) direction of the PC films, as described in [1,8,20]. This technique allows to obtain PC templates with various pore diameter, angle distributio, and porosity characteristics. In the present study, the PC films were subjected to a first irradiation step at two fixed angles of −25${}^{\circ }$ and +25${}^{\circ }$ with respect to the OOP direction of the PC films, with a standard deviation of ±5${}^{\circ }$. After a rotation of 90${}^{\circ }$ in the plane of the PC films, the films were exposed to a second irradiation step at the same fixed angles to form 3D networks of interconnected cylindrical nanochannels, as illustrated in Figure 1a. The PC templates were designed with well-defined pore diameters ϕ in the range of 40 nm to 230 nm, with a dispersion of ±5%, and with different porosity characteristics ranging from P≈ 0.75% to P≈ 20%. For the purpose of growing NWs and NTs by electrodeposition, the PC templates were coated on one surface with a thin metallic Cr/Cu or Cr/Au bilayer cathode using a Pfeiffer 500 e-beam evaporator. The Cr adhesion layer thickness was set between 3 nm to 10 nm. The Cu or Au layer thickness was set to more than three times the nanopores diameter, to ensure a uniform and consistent coverage of the nanopore networks withstanding the electrochemical deposition process, which is required to ensure the growth of crossed nanofibers within the templates. However, the cathode thickness was selected as thin as possible to be easily removable for subsequent characterizations.

The 3D nanoporous templates are then filled by direct electrodepositon from the metallic cathode with a very high degree of replication of the nanochannel networks in spite of the complex interconnected morphology of the 3D nanoporous template [8]. Moreover, the versatility in terms of material composition and nanostructuration enables the fabrication of various 3D architectures. Homogeneous NW networks, as illustrated in Figure 1b, made of Ni, Fe, Co, as well as NiFe and NiCo alloys with controlled alloying composition were deposited in potentiostatic control by applying a constant negative potential to reduce metallic ions contained in home-made electrolyte solution into the pores (see in [16,17,22,36,38] for details). The deposition time was used to ensure a fair filling of the template (typically between 10 to 15 μm) without overfilling. 3D Ni crossed NT networks were grown within the crossed nanopores of PC membranes with diameter of 230 nm using the dealloying method [18]. It uses the spontaneous Cu/Ni core/shell arrangement obtained by electrodeposition from a single sulfamate-based electrolyte solution containing Ni and Cu ions [39,40,41,42,43], using constant deposition potentials ranging from −0.8 V to −1.1 V [18]. Figure 1c shows the reduction curve for the deposition of Cu/Ni crossed nanocable networks at potential of −1 V, where a spontaneous phase separation between Cu and Ni leads to the Cu/Ni–core/shell structure as schematically shown in the inset of Figure 1c. Moreover, the volume ratio of the Cu core and Ni shell can be modified by changing the reduction potential. Next, Ni NTs can be obtained from the Cu/Ni core/shell nanostructures using the electrochemical dealloying method, which consists in the selective and complete electrochemical removal of the Cu core by applying a potential of +0.2 V [18]. This method results in Ni nanotubes with insignificant etching due to the passivation of the Ni shell surface when using sulfamate-based electrolytes [40]. The corresponding electrochemical oxidation curve is displayed in Figure 1d, where the progressive reduction of the electrical current towards zero reflects the etching of the Cu core until its complete removal. This process leads to interconnected Ni NT networks with controllable tube thickness in the range of 10 nm to 100 nm, as displayed in Figure 1e. Finally, interconnected NWs with ferromagnetic metal (FM)/Cu multilayer structure have been grown in the host 3D porous templates for various FM composition (FM = Ni, Co as well as NiCo and NiFe alloys) [14,21,22,36] by pulsed electrochemical deposition from a single home-made electrolyte solution in potentiostatic mode [23,24,25]. The process consists of switching between two different potentials, one being sufficiently low to only reduce Cu, while the second one reducing all species, as illustrated in Figure 1f, to successively stack layers of Cu and FM in the nanopores, as illustrated in Figure 1g. To guarantee a dominant reduction of the ferromagnetic metals at high potential and ensure less than 5% of Cu in the FM layer, the ferromagnetic species concentration in the electrolyte solution were much larger than the Cu concentration. The successive layer thicknesses can be controlled using the pulse duration. For Ni${}_{x}$Co${}_{1-x}$/Cu (0 ≤x≤ 1) and NiFe/Cu NWs, the bilayer thickness was optimized to about 15 nm and 10 nm, respectively, with approximately equal FM and Cu layers thicknesses. As the deposition velocity is faster for higher potential and higher concentration, significantly different pulse duration are used for each applied potential. Figure 1f shows the typical potential and current curves of a pulse electrodeposition of NiCo/Cu NWs. Please note that when the templates are filled with NWs or NTs, the term packing fraction is used instead of the membrane porosity, as the studies focus on the NW and NT networks properties. In general, the template porosity is expected to be larger than the effective network packing fraction because the entire nanopore network may not be completely filled with NWs or NTs. Indeed, during the electrodeposition, some nanopores can be blocked by electrodeposited nanofibers as the electrodeposition rate may not be homogeneous in all nanochannels.

## 3. Results

**Figure 2:**
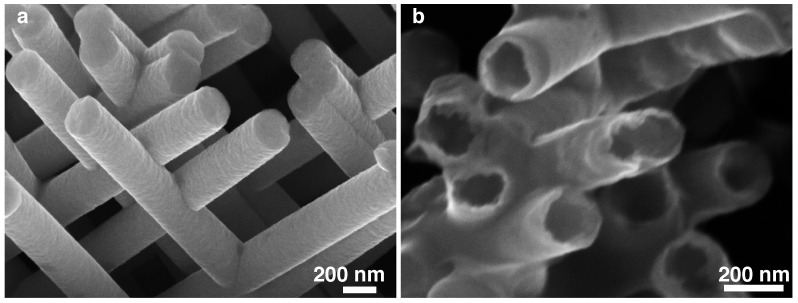
SEM images of (**a**) a Ni crossed nanowire and (**b**) a Ni crossed nanotube networks with 230 nm diameter and 20% of packing fraction, both electrodeposited at a potential of −1 V. The images have been obtained after the complete removal of the polymer host template and testify of the nanowire and nanotube interconnections.

**Figure 3:**
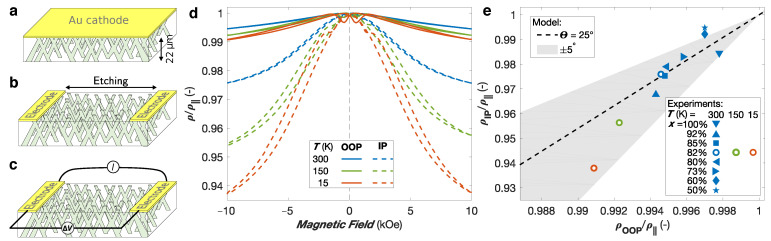
(**a**) Schematic of a 3D interconnected nanowire network electrodeposited from a Au cathode into a 22 μm thick nanoporous polycarbonate film. (**b**) Two-probe electrodes design created by local etching of the cathode. (**c**) Resistance measurement configuration where a current *I* is injected between the two metallic electrodes while recording the voltage differential ∆V induced. (**d**) Anisotropic magnetoresistance curves measured at different temperatures by sweeping an external magnetic field along the out of the plane (OOP; continuous line) and in the plane (IP; dashed line) directions of a permalloy (Ni${}_{82}$Fe${}_{18}$) crossed nanowire network film, deposited in a template with mean pore diameter of 80 nm, ∼3% of porosity. (**e**) Comparison between the model given by Equation (6) for Θ= 25${}^{\circ }$ and the experimental data for nanowire networks (ϕ= 80 nm, P= 3%) made of Ni${}_{x}$Fe${}_{1-x}$ alloys with 0.5 ≤x≤ 1 at room temperature, together with the results at T= 150 K and T= 15 K for x= 0.82. The gray area reflects the calculated relation for Θ in the range 20${}^{\circ }$ to 30${}^{\circ }$.

Figure 2 shows scanning electron microscopy (SEM) images that illustrate the unique feature of the 3D NW and NT networks and provide clear evidence of the NWs and NTs interconnections. The interconnected NW, NT and multilayered NW networks are all found to be robust self-standing structures easily manipulable. Moreover, the tubular feature of the crossed NTs are clearly observable in Figure 2b. Moreover, the networks embedded into the PC template are found to be planar lightweight and flexible films. The interconnections also confer an excellent electrical and thermal connectivity between NWs or NTs that, allowing the easy in-plane measurements of the transport properties. The experimental setups for transport measurements are based on two-probe electrode design created at the surface of the filled template by local plasma etching of the metallic cathode, as shown in Figure 3a–c. To measure the resistance, a current was injected through the electrode design while recording the voltage differential between the two electrodes, as shown in Figure 3c. For this, electrical contacts were directly made on the metallic electrodes using Ag paint. For magneto-transport measurements, a magnetic field was swept between ±10 kOe either along the in-plane (IP) or the OOP directions of the networks films. The magneto-transport measurements were conducted in the temperature range between 10 K and 320 K. It should be noticed that four- and two-probe resistance measurements led to similar results because the electrodeposited NW and NT networks have typical resistance values between few Ω and few kΩ, which are expected to be much larger than the leads and contacts resistance values. For each sample, the input power was maintained below 0.1 μW to avoid self-heating, and the resistance was measured within its ohmic resistance range with a resolution of one part in 10${}^{5}$. Moreover, the measured electrical resistances of the 3D nanofiber networks were found to be unchanged over time, with only slight variation of the resistance (below 1%) when repeating transport experiments even years after the first measurement. Besides, similar magneto-transport properties were obtained on 3D interconnected NW networks when performing the measurement within the polymer template or on the self-standing NW structures after complete chemical dissolution of the polymer template. Finally, it was found that thanks to the flexibility provided by the PC membrane, the network films can be easily twisted without damaging their electrical and magnetic properties.

### 3.1. Anisotropic Magnetoresistance Networks

Figure 3d shows the resistance curves measured at temperatures of 15 K, 150 K, and 300 K with the external magnetic field *H* applied in the OOP and IP directions for an interconnected permalloy (Ni${}_{82}$Fe${}_{18}$) NW network film with 80 nm of diameter and 3% packing fraction. The magnetic field dependencies can be ascribed to the anisotropic magnetoresistance (AMR) effect, which leads to a change in the resistivity of the ferromagnetic metal as the angle between the directions of the magnetization and current directions is modified. As seen in Figure 3d, the maximum resistance is reached near H= 0 for both IP and OOP measurements at all temperatures. This is consistent with remenant magnetization states where magnetization tends to align with the NWs axis when the magnetic anisotropy is dominated by the magnetostatic (MS) contribution [16,17,36]. The parallel configuration between the local current flow and magnetization within the sample leads to a high resistance state [44]. In the saturated state (nearly reached at H = ±10 kOe) of either IP or OOP directions, the magnetization direction is rotated away from the current direction, leading to a decrease in the resistance value. The higher decrease for a magnetic field applied along the IP direction than along the OOP direction is consistent with the mean orientation of the NWs making an angle of 25${}^{\circ }$ with the OOP direction (and thus about 65${}^{\circ }$ with the IP direction). Here, the lower resistance state corresponding to a perpendicular arrangement of the magnetization and the current, $\rho_{⊥}$, cannot be directly measured but can be analytically determined in order to quantitatively estimate the AMR ratio (defined by AMR $=\left(\rho_{\|}-\rho_{⊥}\right)/\rho_{\mathrm{avg}}$, with $\rho_{\mathrm{avg}}=\left(\rho_{\|}+2\rho_{⊥}\right)/3$) [16,17,36]. The analytic model assumes that the resistance state for parallel configuration between the magnetization and the current, $\rho_{\|}$, is reached in absence of magnetic field in 3D NW networks with magnetic anisotropy dominated by the MS contribution, as observed in the AMR curves shown in Figure 3d. In addition, the model assumes that the saturated resistance states in the OOP and IP directions are both reached at H = ±10 kOe, and neglects the NW interconnections contribution due to their modest volume fraction.

Considering a given NW segment with a given orientation θ with respect to the OOP direction, its electrical resistivity depends on the relative orientation between the electrical current flow ***I*** restricted along the NW segment and its magnetization ***M***, that is,

(1) $$\rho \left(\theta \right)=\rho_{⊥}+\left(\rho_{\|}-\rho_{⊥}\right){\left(\frac{I\cdot M}{IM}\right)}^{2}=\rho_{⊥}+\left(\rho_{\|}-\rho_{⊥}\right)\cos^{2}\theta .$$

Because the NWs make an average angle of Θ≈ 25${}^{\circ }$ with respect to the OOP direction (with an estimated variation of ±5${}^{\circ }$), the resistivity at saturation along the OOP and IP directions can be estimated as

(2) $${\bar{\rho }}_{\mathrm{OOP}}=C\rho_{\|}+\left(1-C\right)\rho_{⊥}$$

and

(3) $${\bar{\rho }}_{\mathrm{IP}}=\left(1-C\right)\rho_{\|}+C\rho_{⊥},$$

where $C=\cos^{2}\Theta$. Using $\rho_{\|}$, ${\bar{\rho }}_{\mathrm{OOP}}$, and ${\bar{\rho }}_{\mathrm{IP}}$ as the resistance states at zero field and at H = ±10 kOe in the OOP and IP directions, respectively, the resistance state ($\rho_{⊥}^{\left[x\right]}$, with x= OOP or IP) for the perpendicular configuration between ***M*** and ***I*** can be determined from Equations (2) and (3) as

(4) $$\rho_{⊥}^{\left[\mathrm{OOP}\right]}=\frac{{\bar{\rho }}_{\mathrm{OOP}}-C\rho_{\|}}{1-C}$$

and

(5) $$\rho_{⊥}^{\left[\mathrm{IP}\right]}=\frac{{\bar{\rho }}_{\mathrm{IP}}-\left(1-C\right)\rho_{\|}}{C}.$$

As it is expected that $\rho_{⊥}^{\left[\mathrm{OOP}\right]}=\rho_{⊥}^{\left[\mathrm{IP}\right]}=\rho_{⊥}$, the combination of Equations (4) and (5) gives the expected relation between ${\bar{\rho }}_{\mathrm{IP}}$ and ${\bar{\rho }}_{\mathrm{OOP}}$ as

(6) $${\bar{\rho }}_{\mathrm{IP}}=\frac{C{\bar{\rho }}_{\mathrm{OOP}}+\left(1-2C\right)\rho_{\|}}{1-C}.$$

Figure 3e shows the comparison between Equation (6) and the experimental dependence of ${\bar{\rho }}_{\mathrm{IP}}/\rho_{\|}$ and ${\bar{\rho }}_{\mathrm{OOP}}/\rho_{\|}$ at H = ±10 kOe for crossed NW networks with 80 nm of diameter and 3% in packing fraction made of Ni${}_{x}$Fe${}_{1-x}$ alloys with 0.5 ≤x≤ 1. As seen, the experimental values lie within the grey area that account for the irradiation angle variation of ±5${}^{\circ }$.

**Figure 4:**
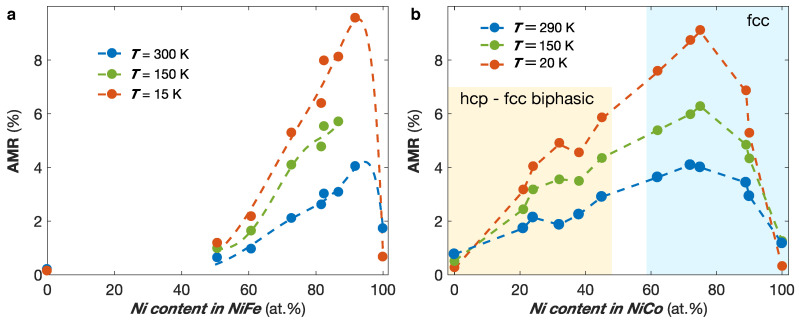
(**a**) Anisotropic magnetoresistance (AMR) ratio as a function of the Ni content *x* at T= 300 K, 150 K, and 15 K for the Ni${}_{x}$Fe${}_{1-x}$, with 0.5 ≤x≤ 1, and Fe nanowire networks with 80 nm in diameter and 3% in packing fraction. The dashed lines are guides for the eyes provided by polynomial approximation of the data. (**b**) AMR ratio as a function of the Ni content *x* at T= 290 K, 150 K, and 20 K for the Ni${}_{x}$Co${}_{1-x}$, with 0 ≤x≤ 1 networks with 40 nm in diameter and 20% in packing fraction. The dashed lines are guides for the eyes. The hcp-fcc biphasic and fcc microstructures of the NiCo alloy nanowires are indicated by the yellow and light blue areas, respectively.

Finally, the AMR ratio of a crossed NW network is expressed as

(7) $${\mathrm{AMR}}^{\left[x\right]}\approx \frac{\rho_{\|}-\rho_{⊥}^{\left[x\right]}}{\rho_{\mathrm{av}}^{\left[x\right]}},$$

where $\rho_{\mathrm{av}}^{\left[x\right]}=1/3\rho_{\|}+2/3\rho_{⊥}^{\left[x\right]}$ is the average resistivity in 3D systems, using x= IP or OOP. No significant differences were found using x= IP or x= OOP to calculate the AMR, as expected. Figure 4a shows the calculated AMR ratio for the Ni${}_{x}$Fe${}_{1-x}$ alloys with 0.5 ≤x≤ 1 at temperatures of 300 K, 150 K, and 15 K. It reveals very large AMR ratios for alloying composition around 90% of Ni, which quickly drop down toward the value of the AMR ratio of Fe for decreasing Ni content, in coherence with previous studies [44,45]. The AMR ratio of Fe NW networks were estimated to about 0.2%, in good agreement with bulk AMR ratio previously measured [46]. Using a similar model, the AMR ratios for Ni${}_{x}$Co${}_{1-x}$ NW networks with 40 nm of diameter and 20% of packing density were estimated for 0 ≤x≤ 1 [17]. The AMR ratio values as a function of *x* at temperatures of 300 K, 150 K, and 20 K are shown in Figure 4b. The high AMR ratio so obtained for Ni${}_{75}$Co${}_{25}$ and the enhancement of the AMR ratio with decreasing temperature for the NiFe and NiCo alloys are in agreement with previous film studies [44,45]. Moreover, the AMR ratio values obtained for interconnected NiCo and NiFe NW networks are also consistent with the values previously reported in arrays of parallel NWs [24,33,34,35].

**Figure 5:**
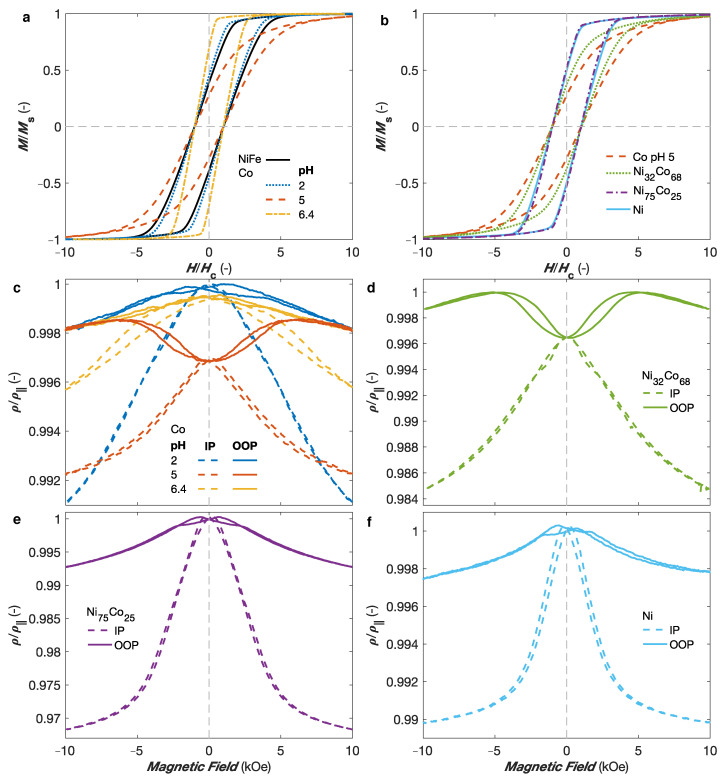
(**a**,**b**) Magnetisation curves as a function of the field ratio $H/H_{c}$, where $H_{c}$ is the corresponding coercive field, recorded while sweeping the external magnetic field in the out-of-plane direction of interconnected nanowire networks with 40 nm in diameter and 20% in packing fraction made of (**a**) NiFe and Co electrodeposited using different electrolytic pH values of 2.0, 5.0, and 6.4 and (**b**) various Ni${}_{x}$Co${}_{1-x}$ alloys, with 0 ≤x≤ 1. (**c**,**f**) Room temperature magnetoresistance curves obtained by applying the magnetic field in the out-of-plane (OOP; full lines) and in-plane (IP; dashed lines) directions for (**c**) the Co nanowire networks in panel (**a**), as well as the (**d**) Ni${}_{32}$Co${}_{68}$, (**e**) Ni${}_{75}$Co${}_{25}$, and (**f**) Ni crossed nanowire networks in panel (**b**).

Studies on Ni${}_{x}$Co${}_{1-x}$ NW networks with 40 nm of diameter and 20% of packing density also revealed the possibility to modify their magnetic anisotropy and magnetoresistance properties using the electrodeposition conditions, material composition and NW networks architecture features [16,17,36,38]. Indeed, in Co and Co-rich NiCo alloys, the possibility to control the microstructures has been shown, allowing to switch from a dominant fcc structure with marginal magnetocrystalline (MC) anisotropy contribution (and thus a dominant MS contribution to the magnetic anisotropy) to an hcp structure with significant MC contribution to the magnetic anisotropy. Moreover, the electrolyte pH of the electrolyte solution is a well-known mechanism to modify the microstructure of Co NWs [47] as well as crossed NWs [16]. Figure 5a shows the room temperature (RT) hysteresis loops recorded while sweeping the external magnetic field along the OOP direction for interconnected Co NW networks electrodeposited at pH 2.0, pH 5.0, and pH 6.4, plotted as a function of the $H/H_{c}$ ratio, with $H_{c}$ their corresponding coercive fields, and compared to a reference Ni${}_{80}$Fe${}_{20}$ crossed NW network with purely MS anisotropy and identical diameter, angle distribution, and packing density characteristics. The coercive field values of the different samples were 944 Oe, 683 Oe, and 1460 Oe for the Co sample deposited at pH 2.0, 5.0, and 6.4, respectively, and 547 Oe for the NiFe sample. Please note that only OOP magnetization curves are reported as the OOP direction has been found to be the easy axis for all these interconnected NW networks [16]. As seen in Figure 5a, the hysteresis loops for the NiFe and Co pH 2.0 samples correspond well one to another, which indicates that they have similar effective magnetic anisotropies arising from MS origin, which is consistent with no favorable structural texture expected for deposition at this pH [16,47]. By opposition, very different magnetic behaviors are found at larger pH values. The significant decrease of the effective magnetic anisotropy for the interconnected Co NWs prepared at pH 5.0 is consistent with the expected textured hcp microsctructure with the c-axis preferentially oriented perpendicularly to the NWs axis, which leads to transverse MC anisotropy contribution competing with the MS contribution; therefore, decreasing the effective anisotropy [16,47]. These changes are accompanied by lower remanence and coercivity in the OOP direction. On the contrary, a very large enhancement of the effective magnetic anisotropy is observed in the interconnected Co NWs prepared at pH value 6.4, for which the hcp c-axis is expected to be preferentially oriented along the NW axis [16,47]. In this case, the MC and MS contributions add, increasing the total anisotropy field. This relationship between the microstructural modification induced by the electrolyte pH and the effective magnetic anisotropy in Co NW networks are in very good agreement with previous measurements performed in arrays of parallel Co NWs [47].

Moreover, the magnetic anisotropy of interconnected NiCo alloy NW networks has been found to depend on the alloying composition [17]. Indeed, for the Ni${}_{x}$Co${}_{1-x}$ (0 ≤x≤ 1) NW networks, a dominant fcc microstructure has been found for x> 45%, while a biphasic hcp-fcc structure with significant transverse MC contribution to the magnetic anisotropy has been obtained for Co-rich NiCo alloys, as illustrated by the light blue and yellow areas in Figure 4b, respectively. Figure 5b shows RT hysteresis loops measured with the external magnetic field applied along the OOP direction of several Ni${}_{x}$Co${}_{1-x}$ NW networks with 0 ≤x≤ 1 plotted as a function of the $H/H_{c}$ ratio. As seen, the Ni and Ni${}_{75}$Co${}_{25}$ samples display similar normalized hysteresis loops than NiFe NWs, reflecting a magnetic anisotropy of MS origin. This contrasts with the hysteresis loops of the Co (pH 5.0) crossed NW network, where the transverse hcp MC contribution to the magnetic anisotropy competes with the MS one, decreasing the OOP effective magnetic anisotropy [17]. The intermediary behavior observed for the Ni${}_{32}$Co${}_{68}$ crossed NW network indicates the presence of a mixture of fcc and hcp phases in Co-rich NiCo NWs, in agreement with the NiCo phase diagram [48,49,50], with the hcp c-axis being predominantly oriented perpendicular to the NW axis, as for the Co NWs deposited at pH 5.0.

Figure 5c shows the magnetoresistance curves measured at RT with the external magnetic field applied along the OOP and IP directions for interconnected Co NW networks deposited at pH 2.0, pH 5.0, and pH 6.4. For the fcc-like Co (pH 2.0) NW network with only MS contribution, the largest resistance state is found close to H= 0, as for the NiFe NW network shown in Figure 3d. However, for the hcp-like Co (pH 5.0) NW network, a clear reduction of the resistance is observed around H= 0, which is indicative of a significant magnetization component transverse to the NW axis in the remenant magnetization state induced by the MC anisotropy contribution, in accordance with the magnetization curves in Figure 5a. As the maximum resistance is not attained at H=0 for this sample, its magnetoresistance curves are normalized by matching the resistance states at H = ±10 kOe in the OOP direction of all Co samples, since the saturated states are expected to be reached in the OOP direction for all Co samples leading to identical resistance states. At higher fields, the resistance state decrease once more as the increasing external magnetic field rotates the magnetization away from the NW axis. As seen in Figure 5c, the saturation in the IP direction is not reached at H = ±10 kOe in the magnetoresistance curves for the Co NW network deposited at pH 6.4, which is also consistent with the hysteresis loops in Figure 5a. This because of the enhanced effective anisotropy along the OOP direction when the MS and MC contributions add. Finally, using the analytic model inherent to the topology of the crossed NW system, the AMR ratio of the Co NW networks have been estimated to about ∼1% [16], which is consistent with the values reported for pure Co [44]. A decrease of the resistance near H= 0 similar to that of the Co pH 5.0 sample has been obtained for the interconnected Co-rich NiCo alloy NWs, as illustrated by the AMR curves for the Ni${}_{32}$Co${}_{68}$ crossed NW network in Figure 5d. The same magnetic behavior has been found on the Co-rich Ni${}_{x}$Co${}_{1-x}$, with x< 45%. Conversely, the maximum resistance state is reached near H= 0, both in the IP and OOP directions, for Ni-rich NiCo samples, as illustrated by the AMR curves for the Ni${}_{75}$Co${}_{25}$ sample in Figure 5e. A similar behavior has been observed for the interconnected Ni NWs as seen in Figure 5f. These AMR curves for Ni and Ni-rich NiCo alloys are consistent with remenant magnetization states where the magnetization lies predominantly parallel to the NW axis because of a dominant MS anisotropy contribution, as also observed in their hysteresis curves in Figure 5b. Similarly, the decrease in resistance at low magnetic field for the Co pH 5.0 and Co-rich NiCo alloys is in agreement with the decrease in the remenant magnetization resulting from the misalignment of the magnetization with respect to the NWs axis, due to the competing MS and MC anisotropies observed for these samples in Figure 5b.

**Figure 6:**
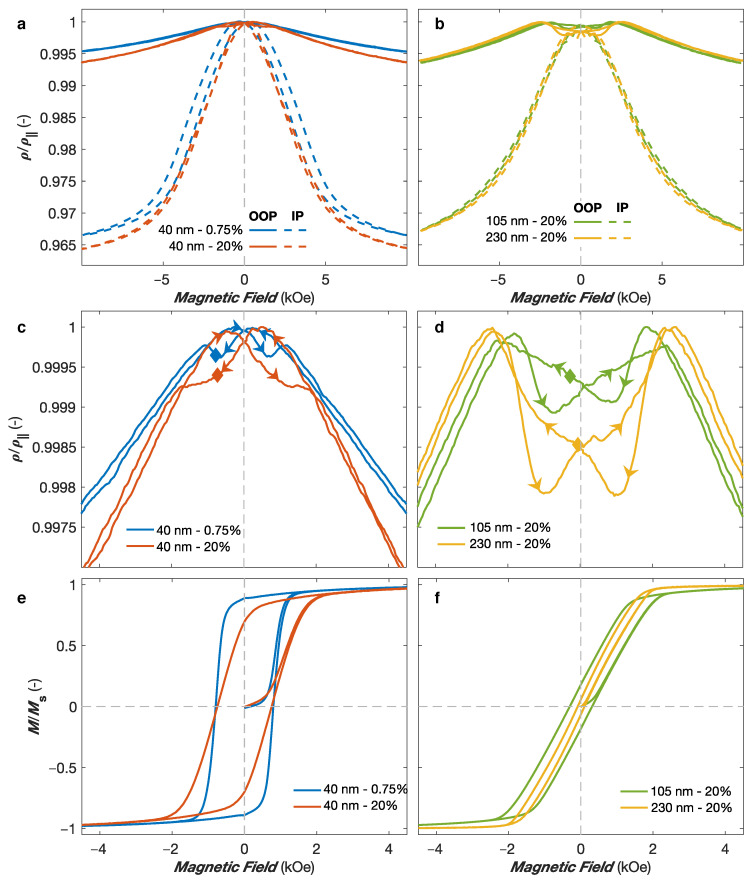
(**a**,**b**) Room temperature anisotropic magnetoresistance curves measured with the external field applied along the out of the plane (OOP; continuous line) and in the plane (IP; dashed line) directions of interconnected Ni${}_{75}$Co${}_{25}$ nanowire networks with (**a**) 40 nm in diameter and ∼0.75% (blue curves) or ∼20% (red curves) in packing density, and (**b**) 105 nm (green curves) and 230 nm (orange curves) in diameter and ∼20% in packing density. (**c**,**d**) Close view of the OOP anisotropic magnetoresistance curves in panels (**a**,**b**) for low magnetic fields. (**e**,**f**) Room temperature hysteresis loops measured with the external field applied along the OOP direction corresponding to panels (**c**,**d**).

Let us now turn to the effect of the diameter and packing density on the magnetic and magneto-transport properties of interconnected NiCo NW networks. Figure 6a,b shows the RT magnetoresistive curves for Ni${}_{75}$Co${}_{25}$ NW networks with 40 nm in diameter and two very different packing density values of ∼0.75% and ∼20% (Figure 6a) and packing density values of ∼20% and diameter of 105 nm and 230 nm (Figure 6b). As seen, similar resistance states are reached at saturation both in the IP and OOP directions. This is expected as the AMR effect is an intrinsic property of the magnetic material and the different samples present similar NWs orientation distributions (∼25${}^{\circ }$ with respect to the OOP direction). The main difference between the samples can be observed at low magnetic fields. Figure 6c,d shows closer views of the OOP magnetoresistance curves at low magnetic fields, which display more noticeable changes. For both NW networks with 40 nm in diameter in Figure 6c, starting from the saturated positive state, a maximum in the resistance is reached at a positive magnetic field. Then, a decrease of the resistance is observed with respect to the global peak shape arising from the magnetization rotation mechanism, and a minimum is reached at the coercive field $H_{c}$ (indicated by full lozenges in Figure 6c). Such behavior has been observed in all the NW networks with purely MS anisotropy with 40 nm in diameter. This finding is consistent with magnetoresistance measurements performed with the magnetic field swept along the NW direction of parallel NW arrays with only MS contribution, where the minimum resistance value is reached when the direction of the magnetic field is reversed, and coincides with the coercive field [33,34,35]. For arrays of parallel NWs, the magnetization reversal mechanism was found to involve the nucleation of magnetic domain walls at the NWs ends that then quickly propagate along the NW axis. Measurements on parallel NW arrays have also found that the angular dependence of the coercive field can be well described by curling (vortex) mode [34,51,52]. In consequence, a similar mechanism is expected in 3D magnetic NW networks with small diameters (40 nm here). The main difference between the NW networks in Figure 6c arises from the very different packing density. The reduction of the packing density is expected to significantly reduce the fraction of NW interconnections with respect to the volume fraction of NWs [14]. Therefore, the low packing density networks is expected to be more similar to the parallel NW system. As seen in Figure 6c, the minimum corresponding to the coercive field is much clearer in the low packing density sample, reflecting a reversal mechanism similar to that of parallel NW arrays (curling mode). For the NW networks with larger diameters, very different behaviors have been found. As seen in Figure 6d, starting from the saturated positive state, the local resistance minima close to zero field are observed at positive field values. Therefore, they do not correspond to the respective coercive fields $H_{c}$ (full lozenges in Figure 6d). For 3D NW networks with large diameter (≥105 nm), the magnetization reversal process is found to be mostly governed by the nucleation of domain walls at the numerous NW interconnections once the external field is reduced, leading to resistance minima observed before reaching H= 0 [18]. Another evidence of that is the effect of the interconnection volume on the reversal mechanisms. As seen in Figure 6d, a more pronounced decrease in resistivity close to H= 0 for larger diameter is observed in the OOP magnetoresistance curves. This means that domain walls with local magnetization mostly oriented perpendicular to the current flow nucleate over a larger volume at the NW segment intersections for larger NW diameter. For smaller diameters (such as 40 nm), the interconnections volume becomes too small to admit domain walls, and the curling reversal mode mechanism becomes dominant.

Figure 6e,f shows the corresponding OOP hysteresis curves at RT for the interconnected Ni${}_{75}$Co${}_{25}$ NW networks. As seen in Figure 6e, the decrease of the packing density generates a higher remanence in the OOP direction because of an increase of the magnetic anisotropy along the OOP direction. This is due to a decrease of the dipolar coupling between the NWs when the packing fraction is reduced [18,38]. For the low packing density sample, the remanence $M_{r}/M_{s}$ reaches about 90%. It should be expected that the low packing fraction NW networks have similar magnetic properties along the OOP direction than arrays of parallel NWs tilted by 25${}^{\circ }$, due to the small volume fraction of NW interconnections, such 3D NW systems being mainly composed of a large number of tilted NW segments. The magnetization curves in the high packing density NW networks in Figure 6e,f show evidence of the decrease of both the remanence and the coercive fields with increasing diameter, as samples with larger diameters are more likely to form multiple domains at the NW crossing zones, due to the demagnetization effect. The decrease in remanence magnetization at zero field for larger NW diameter is in agreement with the decrease in resistance near H= 0 observed in Figure 6c,d resulting from the misalignment between the magnetisation and the current flow. The remanence magnetisation $M_{r}/M_{s}$ decreases from about 70% for the Ni${}_{75}$Co${}_{25}$ NW network with 40 nm in diameter and 20% in packing fraction to approximately 18% and 10% for the Ni${}_{75}$Co${}_{25}$ NW networks with 105 nm and 230 nm in diameter with similar packing fraction, respectively. Similarly, the coercive field $H_{c}$ decreases from about 0.75 kOe for the Ni${}_{75}$Co${}_{25}$ NW network with 40 nm in diameter and 20% of packing density to approximately 0.3 kOe and 0.01 kOe for the Ni${}_{75}$Co${}_{25}$ NW networks with 105 nm and 230 nm in diameter, respectively. The large difference is consistent with a dominant curling reversal mode mechanism for the network with 40 nm in diameter and with a large amount of domain walls in the networks with 105 nm and 230 nm in diameter. Curling mode appears at larger magnetic fields, inducing a larger $H_{c}$ value in the 40 nm sample, and magnetization reversal by curling mode is expected to be insignificant at positive field when starting from the positive saturation state, leading to large remanence magnetization. Conversely, domain walls have weak magnetic properties and can easily be moved, and thus tend to be created at small positive fields, inducing a much smaller remanence in the 105 nm and 230 nm samples.

**Figure 7:**
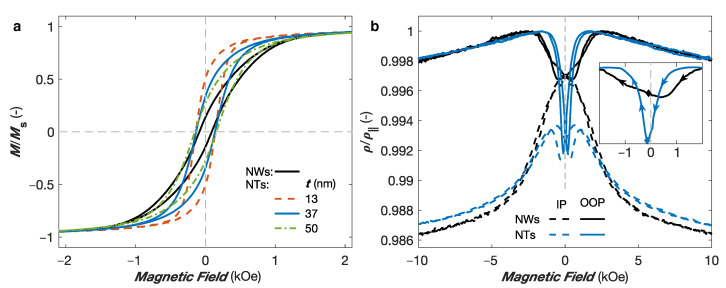
(**a**) Hysteresis loops measured with the magnetic field applied along the out-of-plane (OOP) direction of Ni crossed nanotube networks with 230 nm in diameter and 20% of packing fraction for different wall thicknesses, compared to the hysteresis loop for a Ni crossed nanowire network with similar diameter and packing density characteristics. (**b**) Comparison of the anisotropic magnetoresistance curves measured with the external field applied in the OOP (continuous lines) and in-plane (IP, dashed lines) directions of the crossed NT network with wall thickness of ∼37 nm, and crossed NW networks, both with diameter of 230 nm and packing fraction of about 20%. The inset in panel (**b**) show a zoom at low fields of the OOP AMR curves when sweeping the magnetic field from positive to negative, as indicated by the arrows. The lozenges indicate the resistance states at the corresponding coercive field for each network.

The fabrication of interconnected NT networks with controlled wall thickness is another way to fine-tune the magnetic and magneto-transport properties, as the hollow core add another degree of freedom to modify the magnetic anisotropy [18]. Figure 7a shows the OOP hysteresis loops of several interconnected Ni NT networks with 230 nm in outer diameter, 20% in packing fraction and wall thicknesses of 13 nm, 37 nm, and 50 nm, compared to the OOP hysteresis loops of an interconnected Ni NW network with similar diameter and packing characteristics. As seen, the variation of the NTs wall thickness induces changes in the shape of the hysteresis loops, which is consistent with previous studies on arrays of parallel Ni NTs [39,41]. Notably, the increase of the NTs wall thickness leads to a decrease in the remanence magnetization towards that of the Ni NWs. This remanence reduction can be ascribed to the reduction of the effective magnetic anisotropy due to larger dipolar fields for increasingly closer magnetic charges at the NTs inner surface, in accordance with the decrease of the MS anisotropy due to smaller effective packing fraction of the NT networks [41,53,54]. The much lower remanence values for the Ni crossed NWs arises from much larger dipolar interaction field between NWs, which competes with the demagnetizing field, thus reducing the effective anisotropy field.

Figure 7b compares the AMR curves obtained for two interconnected networks made of Ni NWs and Ni NTs with wall thickness of ∼37 nm, both samples having diameters of 230 nm and packing fraction of 20%. As seen, the resistance state at saturation in the IP and OOP directions are very similar. Moreover, the AMR ratio values obtained using an analytic model for the Ni NW and NT networks were estimated to 1.83% and 1.76%, respectively. These values are consistent with the AMR ratio being an intrinsic property of the magnetic material and correspond well to the AMR ratios previously reported in arrays of parallel Ni NWs [34]. In contrast, different magnetoresistive behaviors between the interconnected Ni NWs and NTs appear at low magnetic fields, in particular along the OOP direction. As seen in the inset of Figure 7b, the OOP AMR curves measured of the Ni NW networks show a decrease in resistivity near H= 0, resulting from the misalignment between the magnetization and the electrical current restricted along the NW segments, which is consistent with the reduced remanence magnetization at zero field observed in the hysteresis curve of the Ni NWs in Figure 7a, with a minimum that does not coincide with the resistance state at coercivity (indicated by a lozenge in the inset of Figure 7b). Starting from the saturated positive state, the minimum in the AMR curve of the Ni NW network is observed at a positive field value. This is consistent with the results obtained in Ni${}_{75}$Co${}_{25}$ NW networks with packing fraction of about 20% and NW diameters of 105 nm and 230 nm in Figure 6d, and can be ascribed to the nucleation of domain walls at the NW interconnections once the external field is decreased. A very different behavior is observed for the interconnected Ni NT network. Starting from the saturated positive state, the OOP AMR curve make a sharp and large peak with a minimum found for a small negative field value that coincides well with the corresponding coercive field (indicated by a lozenge in the inset of Figure 7b). Previous studies revealed that for external magnetic field applied at small angles with respect to the NT axis, the magnetization reversal mechanism is dominated by the curling (vortex) reversal mode with the nucleation and propagation of a large number of vortex domain walls [55,56,57]. This is illustrated by the large decrease in the OOP resistance curves at low magnetic fields shown in Figure 7b, which indicates that the magnetic configuration of the NTs during magnetization reversal account for a larger number of domain walls where the local magnetic moments are mostly perpendicular to the current flow. Please note that the reduction in the resistance curve around the coercive field in the IP configuration observed in Figure 7b can also be attributed to the nucleation of vortex domain walls, as previously suggested from AMR measurements reported for individual Ni NTs [32].

### 3.2. Giant Magnetoresistance Networks

**Figure 8:**
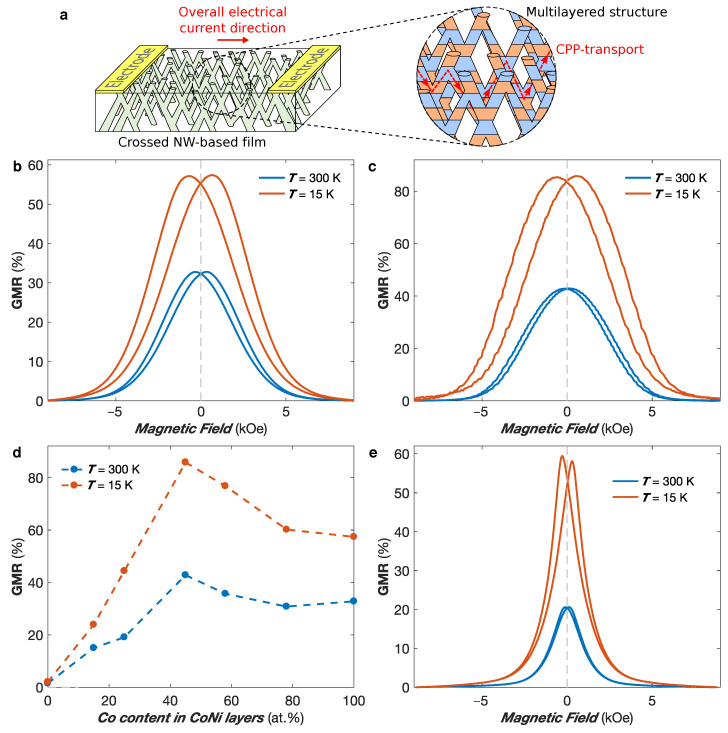
(**a**) Schematic of the giant magnetoresistance measurement in multilayered nanowire-based films, where the electrical transport takes place globally in the plane of the film while the architecture based on crossed nanowires ensures a CPP-type transport. (**b**,**c**) Giant magnetoresistance curves obtained at T= 300 K (blue curves) and T= 15 K (red curves) with the magnetic field applied in the plane of interconnected (**b**) Co/Cu and (**c**) Co${}_{50}$Ni${}_{50}$/Cu nanowire networks with 80 nm in diameter and ∼3% in packing fraction. (**d**) Giant magnetoresistance ratio obtained for interconnected Co${}_{x}$Ni${}_{1-x}$/Cu multilayered nanowire networks with 80 nm in diameter and ∼3% as for 0≤x≤1 at T= 300 K (in blue) and T= 15 K (in red). The dashed lines are guides for the eyes. (**e**) Giant magnetoresistance curves obtained at T= 300 K (blue curves) and T= 15 K (red curves) with the magnetic field applied in the plane of interconnected Ni${}_{80}$Fe${}_{20}$/Cu nanowire networks with 80 nm in diameter and ∼3% in packing fraction.

The successful fabrication of interconnected NW networks made of FM/Cu multilayered NWs makes it possible to measure giant magnetoresistance (GMR) responses with the current flowing perpendicularly to the plane of the layers in the individual NW segments (CPP configuration) and thus overall in the plane of the 3D network film, as illustrated in Figure 8a. This enables the easy measurement of large CPP-GMR effect in flexible multilayered NW networks. These measurements were performed in interconnected FM/Cu NW networks of 80 nm in diameter and 3% in packing fraction, as the reduction of the packing fraction to 3% also greatly reduces the volume fraction occupied by the intersections of the NW segments with respect to the total volume of the NW network. Because the GMR effect is mainly obtained in the NW segments, this reduction of the intersection volume fraction is suitable for producing multilayered structures exhibiting large GMR values. Figure 8b,c shows the CPP-GMR curves at T= 300 K and 15 K obtained by sweeping an external magnetic field in the IP direction of interconnected Co/Cu (Figure 8b) and Co${}_{50}$Ni${}_{50}$/Cu (Figure 8c) NW networks, where the GMR ratio is defined as $\left(\rho \left(H\right)-\rho \left(H_{\mathrm{sat}}\right)\right)/\rho \left(H_{\mathrm{sat}}\right)$, with $H_{\mathrm{sat}}$ the saturation field. For the Co/Cu (Co${}_{50}$Ni${}_{50}$/Cu) sample, the GMR ratio reaches 32.8% (42.8%) at T= 300 K and 57.5% (85.8%) at T= 15 K. Because no significant antiferromagnetic exchange coupling between the successive FM layers are expected for relatively large Cu layer thicknesses (such as the one considered here) [58], the magnetization in the FM layers are expected to be randomly oriented in the remanent state. When an external magnetic field is applied, the FM layer magnetization tend to align along the field direction, which leads to a reduction in the resistance. The GMR ratio values obtained in 3D FM/Cu networks are reported in Table 1 and compared to the values obtained for FM/Cu NWs arranged in parallel arrays. As seen, the values for 3D Co/Cu networks are higher to those previously reported in parallel arrays of Co/Cu NWs [23,58,59,60,61,62], while the low-temperature GMR obtained for the interconnected Co${}_{50}$Ni${}_{50}$/Cu NW network of 85.8% is close to the largest CPP-GMR values recorded in NiCo/Cu multilayers [63]. Please note that these samples display an almost isotropic behavior with very similar magnetoresistive behavior along the IP and OOP directions, which corresponds to the one expected using MS arguments when considering similar magnetic and non-magnetic layer thicknesses for parallel NW arrays [64].

The effect of the NiCo alloying composition on the CPP-GMR responses of Co${}_{x}$Ni${}_{1-x}$/Cu multilayered NW networks with 0≤x≤1 is shown in Figure 8d at RT and T= 15 K. It provides a guideline for the selection of the alloying composition of Co${}_{x}$Ni${}_{1-x}$/Cu multilayers showing large GMR ratios. In particular, a peak in the GMR ratios at both temperatures is observed for equiatomic NiCo alloying composition. An increase of the GMR responses when the temperature is reduced is also clearly observable for all alloying composition, which is consistent with the decrease of the spin mixing effect and the increase of the value of the spin diffusion length with decreasing temperature [65,66]. Figure 8e shows the CPP-GMR curves at T= 300 K and 15 K for a Ni${}_{80}$Fe${}_{20}$/Cu multilayered NW network with the magnetic field applied in the IP direction. The GMR ratio is found to be 20.5% at T= 300 K, which increases almost threefold at T= 15 K (59.4%). As seen in Table 1, the GMR ratio at RT is consistent with the CPP-GMR values reported for parallel Ni${}_{80}$Fe${}_{20}$/Cu NW arrays [67,68]. Moreover, the very large temperature dependence of the GMR is also in agreement with the temperature dependence previously reported in arrays of parallel Ni${}_{80}$Fe${}_{20}$/Cu NWs grown by electrodeposition [24,67,69,70]. Finally, it should be noticed that the saturation field of the Ni${}_{80}$Fe${}_{20}$/Cu NW network is much smaller than the ones of Co/Cu and NiCo/Cu systems deposited in template with identical characteristics. This finding is also in agreement with previous studies on Ni${}_{80}$Fe${}_{20}$/Cu NW arrays [24,67,69,70].

## 4. Conclusions

The present study presented the controlled template-assisted synthesis and characterization in terms of magnetic and magneto-transport properties of 3D ferromagnetic nanowire and nanotube networks. These self-supported nanowire-based nanoarchitectures are found to be mechanically stable. Moreover, the flexibility provided by the polymer membrane and the ease to performed magneto-transport measurements are other advantages of these complex nanostructures. The versatility of the fabrication method allows the control over geometrical features, morphology and chemical composition, which leads to precise control over the magnetic and magneto-transport properties of the network films.

The interplay between the magnetic and magneto-transport properties of 3D nanowire networks made of various ferromagnetic metals and alloys has been explored. The close relationship between the magnetic and structural properties has been found to have a strong influence on the magnetoresistive behaviors of Co and NiCo crossed nanowires. The crystallographic structure of interconnected Co nanowire networks can be modified using the electrolytic bath acidity, which largely impacts their magnetic anisotropy. Large anisotropic magnetoresistance signals (up to 9% at low temperatures), stable over time, were obtained in interconnected NiFe and NiCo nanowire networks, making them interesting nanostructures for applications in 3D magnetic sensing. Moreover, the successful synthesis of crossed nanotube networks with controllable tube wall thicknesses (range: 10–100 nm) have been shown, where the hollow core modifies the magnetic anisotropy and magnetization reversal mechanisms. In addition, giant magnetoresistive responses measured in the current-perpendicular-to-plane configuration in FM/Cu multilayered nanowire network films, with FM = Co, NiCo, or NiFe, up to 43% at RT and 86% at low temperatures have been obtained, showing the possibility to achieve large CPP-GMR responses easily measurable along the macroscopic dimensions of the network films. These unique nano-architectures show great potential as lightweight flexible and shapeable three-dimensional magnetic nanodevices with controlled magnetic anisotropy and spintronics properties for applications such as 3D sensing, data storage, and logic operations, as well as for neuromorphic computing.

## Figures and Tables

**Table 1:** Highest values reported to date of the room temperature giant magnetoresistance ratio for crossed FM/Cu nanowire networks compared to previous measurements on parallel nanowire arrays.

	Co/Cu	Co${}_{50}$Ni${}_{50}$/Cu	Ni${}_{80}$Fe${}_{20}$/Cu
3D networks (this work)	33%	43%	20.5%
Parrallel arrays	23.5% [62]	55% [63]	20% [67]

## Data Availability

The data presented in this study are available on request from the corresponding author.
